# Haematological variables and risk of future venous thromboembolism in the British Regional Heart Study on men. Combined D‐dimer and APTT as a predictive test for thromboembolism?

**DOI:** 10.1111/bjh.18288

**Published:** 2022-06-02

**Authors:** S. Goya Wannamethee, Olia Papacosta, Lucy Lennon, Peter H. Whincup, Ann Rumley, Gordon D. O. Lowe

**Affiliations:** ^1^ Department of Primary Care and Population Health UCL, Royal Free Campus London UK; ^2^ Population Health Research Institute, St George's University of London London UK; ^3^ Institute of Cardiovascular and Medical Sciences University of Glasgow Glasgow UK

**Keywords:** activated partial thromboplastin time, coagulation factor VIII, fibrin D‐dimer, venous thromboembolism

## Abstract

We examined the associations between haematological and inflammatory variables with future venous thromboembolism (VTE), in 3494 men aged 60–79 years, with no previous history of VTE or myocardial infarction, who were not receiving oral anticoagulants. After a mean follow‐up period of 18 years, there were 149 confirmed cases of fatal or non‐fatal VTE (deep vein thrombosis and/or pulmonary embolism). Among classical cardiovascular risk factors, only obesity and cigarette smoking were associated with VTE risk. After adjustment for age, obesity and smoking, VTE risk was associated with coagulation factor VIII, factor IX, von Willebrand factor (VWF), activated partial thromboplastin time (APTT), and fibrin D‐dimer. Hazard ratios (95% CI) for top to bottom quarters (bottom to top for APTT), were respectively 2.17 (1.37, 3.44), 2.15 (1.30, 3.53), 2.02 (1.27, 3.22), 2.43 (1.47, 4.02) and 3.62 (2.18, 6.08). The 11% of men with both the shortest APTT and highest D‐dimer combined had a 5.02 (2.37, 10.62) higher risk of VTE. VTE risk was not associated with fibrinogen, factor VII or activated protein C resistance; full blood count variables or with inflammatory markers, plasma viscosity, C‐reactive protein or interleukin‐6. The combination of D‐dimer and APTT merits evaluation as an adjunct to VTE risk prediction scores.

## INTRODUCTION

Venous thromboembolism (VTE; deep vein thrombosis and pulmonary embolism) is the third most common cause of cardiovascular death in the UK and other developed countries.^1^ Each year, over half a million people die from VTE in the United States and Europe; and 50% of survivors have long‐term complications. Risk factors for VTE include age, male sex, obesity and cigarette smoking; but not diabetes, high blood pressure or blood lipids—in contrast to arterial thrombosis.^2^, ^3^ Half of VTE episodes are provoked in high‐risk medical settings in which routine assessment for VTE risk and antithrombotic prophylaxis can reduce the risk.^4^ For the half of VTE episodes which are non‐provoked, there is a need to establish further risk predictors, in addition to age, obesity and smoking.

Epidemiological studies have identified several prothrombotic risk factors for VTE, including genetic mutations in coagulation factors II and V; and deficiencies in coagulation inhibitors antithrombin, protein C and protein S. The low population frequencies of these mutations make population‐based screening currently impractical in preventing unprovoked VTE.^4^ However, two population cohort studies in the United States (the ARIC and CHS studies, together comprising the longitudinal investigation of thromboembolism aetiology [LITE] study) have reported that high levels of coagulation factors VIII,^5^ IX and XI,^6^ fibrin D‐dimer,^7^, ^8^ and shorter activated partial thromboplastin time (APTT)^9^ are associated with increased risk of future VTE; independently of risk factors and of each other. Zakai et al.^9^ reported interaction between shorter APTT and D‐dimer; and suggested further study of these variables in risk assessment for VTE.

There is a need for further population cohort studies of haematological variables and risk of future VTE,^10^ particularly in older populations at particularly high risk of VTE. In the British Regional Heart Study of older men, we have previously reported the associations of a range of haematological and inflammatory variables with risk of future arterial thrombosis: myocardial infarction^11^ and stroke.^12^ In this study we report their associations with risk of future VTE.

## METHODS

The British Regional Heart Study is a prospective study involving 7735 men aged 40–59 years drawn from one general practice in each of 24 British towns, who were screened between 1978 and 1980.^13^ The population studied was socioeconomically representative of British men and comprises predominantly white Europeans (>99%). In 1998–2000, all surviving men, then aged 60–79 years, were invited for a 20th year follow‐up examination. All men completed a mailed questionnaire providing information on their lifestyle, medical history and medication; had a physical examination and provided a fasting blood sample. 4252 men (77% of survivors) attended for examination. A total of 4088 men had at least one haematological or inflammatory marker measured. We excluded men with baseline doctor diagnosed myocardial infarction or a history of a diagnosis of deep vein thrombosis or pulmonary embolism, and all men on anticoagulant drugs (*n* = 594). After these exclusions, 3494 men were available for analysis.

### Cardiovascular risk factors

Details of measurement and classification methods for body mass index (BMI; weight/height^2^), waist circumference (WC), smoking status, physical activity, social class, alcohol intake, blood pressure and blood lipids have been previoiusly described.^14^ Prevalent diabetes included men with a diagnosis of diabetes, or fasting blood glucose ≥7 mmol/l.

### Haematological and inflammatory variables

At the 20‐year examination, blood was anticoagulated with K_2_ EDTA (1.5 mg/ml) for measurement of full blood count (Coulter S), and plasma viscosity at 37°C in a semi‐automated capillary viscometer (Coulter Electronics). Blood was also anticoagulated with 0.109 M trisodium citrate (9:1 v:v) for measurement of clottable fibrinogen (Clauss method); as well as coagulation factors VII, VIII and IX; activated partial thromboplastin time (APTT) and activated protein C (APC) ratio (as a measure of APC resistance) in an MDA‐180 coagulometer (Organon Teknika). Fibrin D‐dimer was measured with an enzyme‐linked immunosorbent assay (Biopool AB) as was von Willebrand factor (VWF) antigen (Dako). C‐reactive protein (CRP) was assayed by ultra‐sensitive nephelometry (Dade Behring). Interleukin‐6 (IL‐6) was assayed using a high‐sensitivity ELISA (R & D Systems). Distributions and laboratory coefficients of variation have been described previously.^12^

### Follow‐up

All men have been followed up from initial examination (1978–1980) for cardiovascular morbidity and mortality and follow‐up has been achieved for 99% of the cohort.^13^ In the present analyses, all‐cause mortality and morbidity events are based on follow‐up from re‐examination in 1998–2000 at mean age 60–79 years to June 2018. Survival times ended at the first VTE event or when participants were censored for death due to any cause, or the end of the follow‐up period, whichever occurred first. Information on death was provided by the UK National Health Service registers. Incident VTE included fatal and non‐fatal VTE. Non‐fatal VTE was based on a doctor‐confirmed diagnosis of VTE from primary care medical records (including hospital and clinical correspondence). All cases were verified by a review of available clinical information from primary and secondary care records (symptoms, signs, investigations, and treatment response) considering information on ultrasound scans, venograms, D‐dimer measurements, lung scans and pulmonary angiograms where available. Incident fatal VTE cases were those in which the diagnosis of VTE was mentioned as the underlying cause of death in death certificates. Fatal VTE events were defined as ICD ninth revision, codes 451 and 453 (DVT) and 415 (PE).

### Statistical analysis

Analyses are based on the division of coagulation variables into quartiles. Chi‐squared tests were used to test the difference in the distribution of baseline characteristics between those who developed VTE and those who did not. The *t*‐test was used to test the differences in mean levels of risk factors between the two groups. Cox's proportional hazards model was used to assess the multivariate‐adjusted relative risk for the highest quartile compared with the lowest quartile (reference group). For APTT the reference group was the highest quartile for comparisons with other factors. In the adjustment, smoking (long term ex‐smokers [>15 years], recent ex‐smokers and current smokers) was fitted as a categorical variable. BMI was fitted as a continuous variable. Tests for trends were carried out fitting the coagulation variables in their original continuous form. To identify the best predictive models, the areas under the curve (AUC) (c‐statistics) were used to assess the ability of blood markers to predict VTE beyond a score which included conventional routine risk factors age, BMI and smoking. Finally, analyses were performed for combinations of tertiles of D‐dimer and APTT.

## RESULTS

During the mean follow‐up period of 18 years (total 48 670 person‐years of analysis), there were 149 incident VTE cases in the 3494 men with no history of MI or VTE and who were not on anticoagulant drugs.

### Baseline characteristics

Table 1 compares classical cardiovascular risk factors, and haematological, haemostatic and inflammatory variables in men with and without incident VTE. Those who developed VTE had higher BMI and larger waist circumference than those who did not develop VTE, but they were less likely to be current smokers and more likely to have been recent ex‐smokers. D‐dimer and coagulation factors VIII, IX, VWF were significantly higher, and APTT significantly shorter, in those who developed VTE. No significant difference was seen in levels of other haematological variables, or inflammatory markers, between those who developed VTE and those who did not.

**TABLE 1:** Distributions of baseline demographics, cardiovascular risk factors and haematological or inflammatory variables, according to venous thromboembolism (VTE) status at follow‐up

	No incident VTE (*N* = 2848)	Incident VTE (*N* = 148)	*p*‐difference between groups^a^
CVD risk factors			
Age (years)	68.6 (5.49)	68.0 (5.24)	0.27
BMI (kg/m^2^)	26.77 (3.69)	27.56 (3.61)	0.009
WC (cm)	96.8 (10.27)	99.54 (9.61)	0.002
% Inactive	9.8	11.5	0.49
% Current smokers	13.2	6.1	0.01
% Recent ex‐smokers	19.3	26.4	0.03
% Diabetes	12.7	10.8	0.51
% Manual workers	53.4	55.4	0.63
% Heavy drinkers	3.8	4.7	0.56
Total cholesterol (mmol/l)	6.04 (1.06)	6.04 (1.04)	0.96
Systolic blood pressure (mmHg)	150.3 (23.9)	146.7 (24.2)	0.08
HDL cholesterol (mmol/l)	1.33 (0.34)	1.30 (0.33)	0.27
Blood group (%)			
Non‐O	52.0	58.1	0.13
O	48.0	41.9	
A	40.2	44.2	
AB	9.2	10.2	
B	2.6	2.0	
Coagulation factors			
Log D‐dimer (ng/ml)	4.39 (0.82)	4.63 (0.80)	<0.0001
Factor VII (iu/dl)	118.2 (25.47)	116.8 (23.65)	0.52
Factor VIII (iu/dl)	131.3 (31.35)	138.3 (33.55)	0.007
Factor IX (iu/dl)	131.5 (25.06)	136.6 (24.41)	0.01
VWF (iu/dl)	138.0 (46.04)	145.7 (42.47)	0.04
APTT (s)	30.97 (3.59)	29.96 (3.02)	0.005
APC ratio	3.28 (0.55)	3.29 (0.59)	0.93
Fibrinogen (g/l)	3.26 (0.73)	3.16 (0.70)	0.12
Inflammation markers			
Log CRP (g/l)	0.52 (1.11)	0.66 (1.03)	0.12
Log IL‐6 (pg/ml)	0.88	0.84	0.42
Plasma viscosity (mPa.s)	1.28 (0.08)	1.28 (0.11)	0.93
Full blood count			
WBC count ×10^9^/l	1.92 (0.27)	1.90 (0.31)	0.36
RBC count ×10^12^/l	4.86 (0.40)	4.85 (0.39)	0.58
Platelet count ×10^9^/l	235.9 (62.7)	235.7 (67.4)	0.96
MPV fl	8.40 (1.71)	8.19 (1.70)	0.13
MCH pg	30.08 (1.83)	30.0 (2.18)	0.36
MCHC g/l	0.32 (0.01)	0.32 (0.01)	0.39
MCV fl	92.9 (5.25)	92.9 (5.76)	0.98
Haematocrit l/l	45.13 (3.38)	44.93 (3.30)	0.49
Haemoglobin g/l	14.60 (1.18)	14.50 (1.17)	0.31

Abbreviations: MCH, mean corpuscular haemoglobin; MCHC, mean corpuscular haemoglobin concentration; MCV, mean corpuscular volume; MPV, mean platelet volume; RBC, red blood cell; WBC, white blood cell; WC, waist circumference.

^a^ Chi‐squared and *t*‐tests to obtain *p*‐values for difference in prevalence and mean levels between the two groups, respectively.

### Coagulation variables and incident VTE

In age adjusted analyses, D‐dimer, factor VIII, IX, VWF and APTT were positively associated with risk of incident VTE. Table 2 shows the adjusted hazard ratio (95% CI) for incident VTE for these variables. They remained significantly associated with risk of VTE after adjustment for age, BMI and smoking status. Many were significantly correlated (Table 3) and the association between factor IX and risk of incident VTE was markedly attenuated after adjustment for factor VIII (Table 2). By contrast, factor VIII remained significantly associated with incident VTE after adjustment for factor IX (HR, top quartile vs. bottom quartile 1.74 [1.04, 2.90]; *p* = 0.02 for trend). Factor VIII and VWF were highly correlated (r = 0.70) and when both were included in the model each became non‐significant, although factor VIII showed stronger associations with VTE compared to VWF (1.60 [0.90, 2.86], *p* = 0.09 compared to 1.40 [0.77, 2.54], *p* = 0.31). Adjustment for D‐dimer made little difference to the association between APTT and VTE; the associations of VTE with factor VIII and with factor IX were partially attenuated, but each remained significant.

**TABLE 2:** Coagulation variables and adjusted hazards ratio, HR (95% CI) for incident VTE, per quartile

	Age adjusted	Model 1	Model 1 + FVIII	Model 1 + D‐dimer
D‐dimer				
1	1.00	1.00	1.00	
2	1.21 (0.69, 2.14)	1.22 (0.69, 2.16)	1.16 (0.66, 2.05)	‐
3	2.29 (1.36, 3.85)	2.20 (1.31, 3.71)	2.04 (1.20, 3.46)	‐
4	3.62 (2.18, 6.01)	3.57 (2.14, 5.94)	3.19 (1.90, 5.37)	‐
Trend	*p* < 0.0001	*p* < 0.0001	*p* < 0.0001	
Factor VIII			Model 1 + VWF	
1	1.00	1.00	1.00	1.00
2	1.12 (0.67, 1.87)	1.05 (0.63, 1.76)	0.98 (0.58, 1.65)	0.97 (0.58, 1.65)
3	1.53 (0.94, 2.49)	1.45 (0.89, 2.35)	1.27 (0.75, 2.15)	1.31 (0.81, 2.13)
4	2.17 (1.37, 3.44)	2.01 (1.27, 2.18)	1.60 (0.90, 2.86)	1.70 (1.07, 2.71)
Trend	*p* = 0.0002	*p* = 0.0008	*p* = 0.09	*p* = 0.01
Factor IX			Model 1 + FVIII	
1	1.00	1 .00	1.00	1.00
2	1.44 (0.86, 2.42)	1.43 (0.84, 2.43)	1.30 (0.76, 2.22)	1.42 (0.83, 2.40)
3	1.77 (1.06, 2.95)	1.76 (1.05, 2.97)	1.50 (0.88, 2.57)	1.72 (1.02, 2.89)
4	2.15 (1.30, 3.53)	2.02 (1.20, 3.38)	1.55 (0.88, 2.72)	1.85 (1.10, 3.11)
Trend	*p* = 0.003	0.01	*p* = 0.27	*p* = 0.03
APTT			Model 1 + FVIII	
1	2.43 (1.47, 4.02)	2.49 (1.48, 4.45)	2.09 (1.21, 3.59)	2.31 (1.38, 3.86)
2	1.96 (1.16, 3.31)	1.99 (1.17, 3.39)	1.81 (1.05, 3.11)	1.92 (1.13, 3.28)
3	1.29 (0.74, 2.25)	1.34 (0.76, 2.34)	1.29 (0.73, 2.26)	1.33 (0.76, 2.34)
4	1.00	1.00	1.00	1.00
Trend	*p* = 0.0002	*p* = 0.0001	*p* = 0.004	*p* = 0.0006
VWF			Model 1 + FVIII	
1	1.00	1.0	1.00	1.00
2	1.07 (0.64, 1.78)	1.04 (0.60, 1.67)	0.94 (0.55, 1.58)	0.98 (0.59, 1.64)
3	1.61 (1.01, 2.59)	1.51 (0.94, 2.43)	1.26 (0.75, 2.11)	1.39 (0.87, 2.24)
4	2.02 (1.27, 3.22)	1.93 (1.21, 3.08)	1.40 (0.77, 2.54)	1.65 (1.03, 2.64)
Trend	*p* = 0.0007	*p* = 0.002	*p* = 0.26	*p* = 0.03

*Note*: Model 1 = adjusted for age, BMI and smoking.

**TABLE 3:** Correlations between coagulation variables

	D dimer	FVIII	FIX	VWF	APTT
D‐dimer	1.00	0.25	0.15	0.28	−0.09
Factor VIII		1.00	0.43	0.70	−0.33
Factor IX			1.00	0.20	−0.43
VWF				1.00	−0.14
APTT					1.00

*Note*: All *p* < 0.0001.

Table 4 shows the associations between D‐dimer, factor VIII and APTT with incident VTE, adjusting for age, BMI, smoking and each of the other coagulation variables. D‐dimer showed the strongest association, followed by APTT. The association seen for factor VIII was markedly attenuated after adjustment for APTT.

**TABLE 4:** Quartiles of D‐dimer, factor VIII, APTT and HR (95%CI) for incident VTE, after adjustment for age, BMI, smoking and each of the other variables

	HR (95%CI)
D‐dimer	
1	1.00
2	1.16 (0.65, 2.05)
3	2.00 (1.18, 3.39)
4	3.13 (1.86, 5.27)
Trend	*p* < 0.0001
Factor VIII	
1	1.00
2	0.88 (0.52, 1.40)
3	1.08 (0.66, 1.79)
4	1.30 (0.79, 2.14)
Trend	*p* = 0.22
APTT	
1	2.06 (1.20, 3.56)
2	1.81 (1.05, 3.12)
3	1.30 (0.74, 2.28)
4	1.00
Trend	*p* = 0.006

To identify the best predictive models in predicting VTE we obtained c‐statistics for models with and without biomarkers (D‐dimer, APTT and factor VIII). Table 5 shows the c‐statistics for the model with age, BMI and smoking and the improvement in C‐statistics in models with blood markers D‐dimer, APTT and factor VIII. A simple model with age, smoking and BMI yielded a c‐statistics of 0.575 (0.529, 0.593). Adding both D‐dimer and APPT improved prediction significantly (c statistic = 0.657 [0.613, 0.701]). By contrast adding factor VIII to the model made no improvement in the c‐statistic (c = 0.653 [0.609, 0.697]). Thus, a model which included age, BMI, smoking, D‐dimer and APPT provided the best c‐statistics.

**TABLE 5:** C‐statistics (95%CI) for clinical models and improvement in c‐statistics for conventional model (age, BMI and smoking) with D‐dimer, APTT and factor VIII

Model	C‐statistics	*p*‐value improvement	
1. Age, BMI and smoking	0.575 (0.529, 0.620)		‐
2. Age, BMI, smoking +D‐dimer	0.631 (0.529, 0.673)	Model 2 vs. Model 1	*p* = 0.007
3. Age, BMI, smoking +APTT	0.629 (0.583, 0.674)	Model 3 vs. Model 1	*p* = 0.006
4. Age, BMI, smoking+D‐dimer+APTT	0.657 (0.613, 0.701)	Model 4 vs. Model 2	*p* = 0.04
5. Age, BMI, smoking+APTT+D‐dimer+Factor VIII	0.653 (0.609, 0.697)	Model 5 vs. Model 4	*p* = 0.50

### Combined effect of D‐dimer and APTT on risk of VTE

**FIGURE 1:**
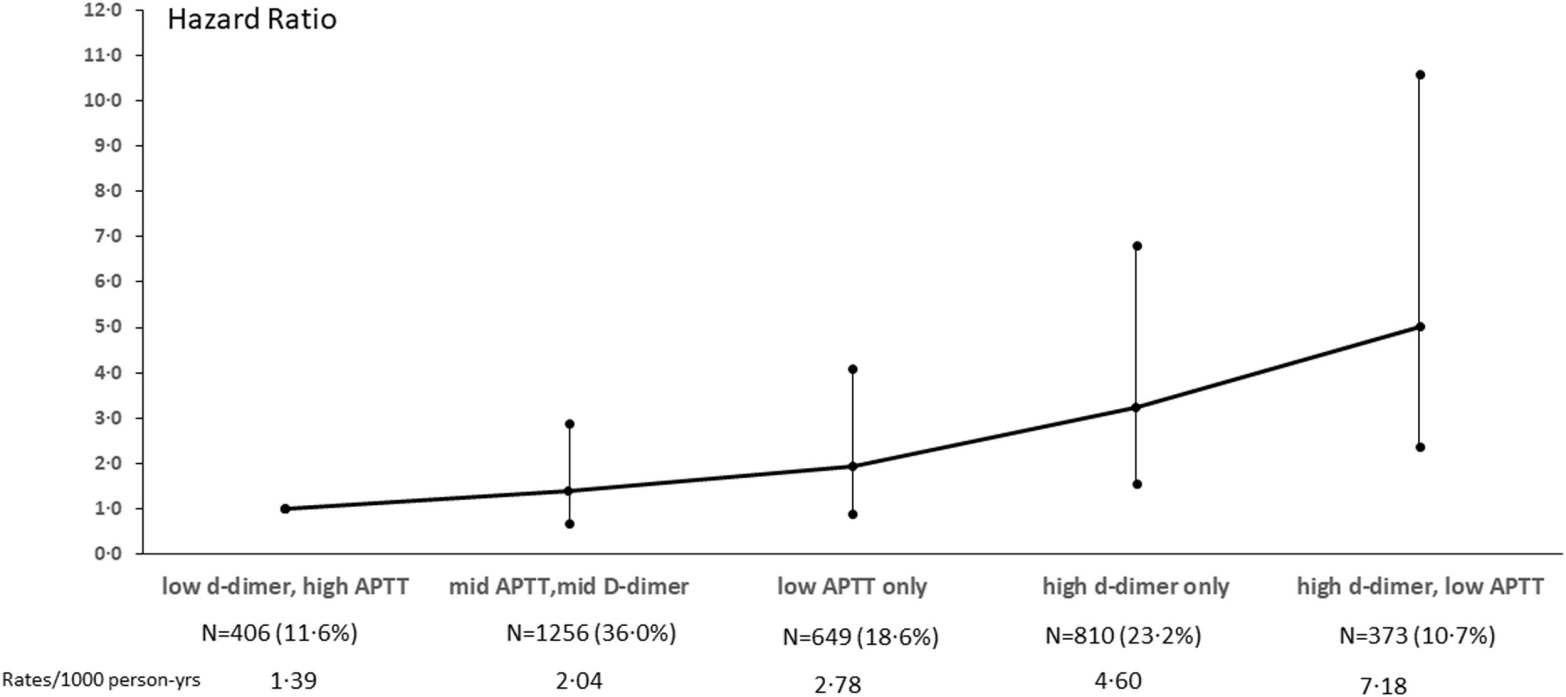
Combinations of tertiles of APTT and D‐dimer and associations with incident VTE, adjusted for age, BMI and smoking. Low, lowest tertile; mid, middle tertile; high, top tertile. Tertiles of APTT: <29.2, 29.2–31.6, ≥31.7. Tertiles of D‐dimer: <57, 57–99.9, ≥100.

To assess the combined influence of APTT and D‐dimer on risk of VTE and to achieve sufficient numbers, we stratified the APTT and D‐dimer by tertiles of the distribution. Figure 1 shows the combined analysis of D‐dimer and APTT and the hazard ratio (95%CI) for incident VTE, adjusted for age, BMI and smoking. The lowest risk of VTE was in the 12% of men in the lowest tertile of D‐dimer, and the highest tertile of APTT. Compared to that group, the 36% of men in the middle (33.3–66.6) tertiles of both D‐dimer and APTT had a non‐significantly higher risk of VTE: HR 1.40 (0.67, 2.90). The risk of VTE increased to two‐ fold in those with low APTT only, three‐fold in those with high D‐dimer only and to five‐fold in the 11% of men with both high D‐dimer and low APTT: HR 5.02 (2.37, 10.62). Among half the men (47.6%) who did not fall into either the lowest tertile of APTT or highest tertile of D‐dimer (bottom 2 stratum) the absolute risk was 1.9/1000 person years. Compared to this larger group of men the HR (95%CI) for those with low APTT only, elevated D‐dimer only and those with low APTT and high D‐dimer were 1.48 (0.92, 2.309), 2.50 (1.63, 3.83) and 3.86 (2.43, 6.11), respectively.

## DISCUSSION

We report the first large UK prospective population‐based study of a range of haematological variables and risk of future VTE, within a representative sample of older men from across the UK. We confirm the findings in two prospective population studies from the United States (the ARIC and CHS studies, comprising the LITE study) that circulating concentrations of coagulation factor VIII,^5^ fibrin D‐dimer,^7^, ^8^ and short APTT^9^ are associated with increased risk of VTE; independently of classical CVD risk factors and of each other.

We report that D‐dimer showed the strongest association with VTE, independent of age, BMI, smoking and other coagulation variables. This confirms previous studies.^8^, ^15^

Factor VIII and VWF circulate as a high‐affinity non‐covalent complex,^16^ and their plasma levels are highly correlated in general population sample: r = 070 in our study. We observed similar associations of factor VIII and VWF level with risk of future VTE. When both were included in the model their associations with incident VTE were, unsurprisingly, attenuated, although factor VIII showed the stronger association. The LITE study reported that FVIII and VWF were associated with VTE risk independently of each other.^5^ While case–control studies including LETS^17^ and MEGA^18^ confirmed similar associations of factor VIII and VWF with VTE risk, on multivariate analysis they did not show associations independently of each other: similar to the current study.

A direct causal effect of the factor VIII/VWF complex on risk of VTE is supported by a systematic review and meta‐analysis of studies of ABO(H) blood groups non‐O, which confer higher levels of factor VIII and VWF than group O.^19^ This reported a pooled odds ratio of 1.79 (95% CI 1.56, 2.05) for non‐O blood group for VTE. The LITE study reported that ABO blood group and factor VIII were independently associated with risk of VTE.^20^ In the present study, we found that non‐O blood group was non‐significantly associated with risk of VTE: hazard ratio for non‐O versus O 1.28 (0.92, 1.77).

Our study appears to be the first population‐based cohort study of incident VTE and factor IX level. Factor IX was associated with risk of future VTE; but this was attenuated by adjustment for factor VIII. Population‐based case–control studies have shown variable results.^6^, ^18^, ^21^, ^22^, ^23^ The largest study, MEGA,^18^ which included all coagulation factors, showed that while factor VIII and VWF had the largest effect on VTE risk, factor IX (and factor XI) showed weaker, but significant associations.

The present study confirms the findings of the LITE study that shortened APTT is independently associated with risk of future VTE, after adjustment for obesity, factor VIII, factor IX, and D‐dimer; the LITE study also adjusted for factor XI.^9^ Similar results on APTT were reported from a case–control study of VTE.^24^ In the prospective, multicentre ECAT‐DVT study of many haemostatic variables and risk of DVT after elective hip replacement, APTT was the only one to show an independent predictive value for DVT risk.^25^

Risk of future VTE was not associated with fibrinogen or factor VII; consistent with reports from the LITE cohort study^4^, ^8^; and from the large MEGA study of all coagulation factors.^18^ We found no association of activated protein C resistance, measured as APC ratio, with risk of VTE. This is likely due to its strong association with the factor V Leiden mutation, which has a low prevalence in the UK population of 2.5%.^26^

Risk of VTE was not associated with full blood count variables; nor with the inflammatory markers plasma viscosity, C‐reactive protein or interleukin‐6. The lack of associations with inflammatory markers is consistent with reports from the LITE cohort study,^5^ and the Malmo cohort study.^27^

Our findings showed that when FVIII, APTT and D‐dimer were included in the same model, hazard ratios remained significant for D‐dimer and lower APTT; but not for FVIII. This finding suggests that APTT captures the effect of FVIII on VTE risk, which may reflect complex interactions of coagulation system proteins not captured by individual factor assays.^10^

The most important finding from our study is that it prospectively confirms the suggestion of Zakai et al.^9^ that the combination of D‐dimer and lower APTT identifies men at highest risk of VTE. The lowest risk of VTE was in men with low (lowest tertile) D‐dimer and high (top tertile) APTT. Compared to this group those with low APTT alone or high D‐dimer alone showed a two‐to‐three‐fold increase in risk even after taking age, BMI and smoking into account and this increased to five‐fold in men with both high D‐dimer and low APTT. Compared to the 47.6% of men who did not have low APTT or high D‐dimer, the risk of VTE in those in the top risk category (low APTT and high D‐dimer) was increased over three‐fold.

The predictive value of clinical variables for prediction of VTE in the UK can be assessed by the Qthrombosis score (Qthrombosis.org). For an average male non‐smoker aged 70 years, with average BMI of 26 kg/m^2^, the 5‐year risk of VTE is about 1.5%. We estimate that this average risk would be more than trebled in the 11% of men who are in both the top third of D‐dimer levels, and the bottom third of APTT: increasing the 5‐year risk of VTE to about 5%. Identification of this level of risk might have preventive value: either focussed lifestyle advice (reducing weight and smoking habit, increased activity), or consideration of antithrombotic prophylaxis.^4^ It could also identify this high risk 11% of the population in whom randomised controlled trials of low dose direct anticoagulant treatment might be performed.

Thus, it may be feasible to combine APTT and D‐dimer in risk assessment for VTE. Both tests are routinely performed in UK haematology laboratories, to assess bleeding risk and VTE risk; and are included in a national quality assessment schemes (NEQAS Blood Coagulation). Our study was performed on stored frozen plasma samples from citrated blood, demonstrating that their assay in batches provides relevant data to inform individual risk assessment.

Our cohort of older men in the BRHS (men aged 60–79 years) are at high risk of a first episode of VTE. Half of all population VTE cases occur in this age group, and the risk is significantly higher in men than women.^28^ We have shown in BRHS men significant increases in FVIII and D‐dimer with increasing age in this group,^29^ which from this report may be plausible contributors to their increased risk of VTE. We found no association of APTT with age. While our population sample is representative of the UK population, there is a need to repeat our study of VTE risk factors in women.

Strengths of our study include a large representative cohort sample of the older UK male population who are at high risk of future VTE; and a comprehensive assessment of haematological and inflammatory variables which may be markers of VTE risk. Limitations include a largely white European male sample. Further population studies of FVIII, FIX, VWF, D‐dimer and APTT and risk of VTE are required, and in due course meta‐analyses to establish the strength of these associations, and their potential in risk prediction when added to classical risk factors.

## AUTHOR CONTRIBUTIONS

GDOL and SGW initiated the concept and design of the paper. SGW analysed the data. GDOL and SGW drafted the manuscript. PHW contributed to the interpretation of data. OP contributed to the analysis of the paper. LL and PHW contributed to the acquisition of the data. AR supervised the laboratory analyses. All authors revised the manuscript critically for important intellectual content and approved the final version.

## FUNDING INFORMATION

The British Regional Heart Study is a Research Group supported by the British Heart Foundation (BHF) Programme grant (RG/19/4/34452). Analyses of haematological and inflammatory variables were supported by British Heart Foundation Project grants.

## References

[1] Di Nisio M , van Es N , Büller HR . Deep vein thrombosis and pulmonary embolism. Lancet. 2016;388(10063):3060–73. 27375038 10.1016/S0140-6736(16)30514-1

[2] Mahmoodi B , Cushman M , Naess IA , Allison MA , Bos WJ , Brækkan SK , et al. Association of cardiovascular risk factors with venous thromboembolism: an individual participant data meta‐analysis of published studies. Circulation. 2017;135:7–16. 27831499 10.1161/CIRCULATIONAHA.116.024507 PMC5201424

[3] Gregson J , Kaptoge S , Bolton T , Pennells L , Willeit P , Burgess S , et al. Cardiovascular risk factors associated with venous thromboembolism. JAMA Cardiol. 2019;4:163–73. 30649175 10.1001/jamacardio.2018.4537 PMC6386140

[4] Folsom A , Cushman M . Exploring opportunities for primary prevention of unprovoked venous thromboembolism: ready for prime time? J Am Heart Assoc. 2021;9:e019395. 10.1161/JAHA.120.019395 PMC7763794 33191841

[5] Tsai A , Cushman M , Rosamond WD , Heckbert SR , Tracy RP , Aleksic N , et al. Coagulation factors, inflammation markers, and venous thromboembolism: the longitudinal investigation of thromboembolism etiology (LITE). Am J Med. 2002;113:636–42. 12505113 10.1016/s0002-9343(02)01345-1

[6] Cushman M , O'Meara ES , Folsom AR , Heckbert SR . Coagulation factors IX through XIII and the risk of future venous thrombosis: the longitudinal investigation of thromboembolism etiology. Blood. 2009;114:2878–83. 19617576 10.1182/blood-2009-05-219915 PMC2756198

[7] Cushman M , Folsom AR , Wang L , Aleksic N , Rosamond WD , Tracy RP , et al. Fibrin fragment D‐dimer and the risk of future venous thrombosis. Blood. 2003;101:1243–8. 12393393 10.1182/blood-2002-05-1416

[8] Folsom AR , Alonso A , George KM , Roetker N , Tang W , Cushman M . Prospective study of plasma D‐dimer and incident venous thromboembolism: the atherosclerosis risk in communities (ARIC) study. Thromb Res. 2015;136:781–5. 26337932 10.1016/j.thromres.2015.08.013 PMC4577468

[9] Zakai NA , Ohira T , White R , Folsom AR , Cushman M . Activated partial thromboplastin time and risk of future venous thromboembolism. Am J Med. 2008;121:231–8. 18328308 10.1016/j.amjmed.2007.10.025 PMC2295205

[10] Lowe GDO . Epidemiology of venous thromboembolism: the need for large (including prospective) studies and meta‐analyses. J Thromb Haemost. 2012;10:2186–8. 22888854 10.1111/j.1538-7836.2012.04882.x

[11] Wannamethee SG , Whincup PH , Shaper AG , Rumley A , Lennon L , Lowe GDO . Circulating inflammatory and hemostatic biomarkers are associated with risk of myocardial infarction and coronary death, but not angina pectoris, in older men. J Thromb Haemost. 2009;7:1605–11. 19682232 10.1111/j.1538-7836.2009.03574.x PMC2810437

[12] Wannamethee SG , Whincup PH , Lennon L , Rumley A , Lowe GD . Fibrin D‐dimer, tissue‐type plasminogen activator, and risk of incident stroke in older men. Stroke. 2012;43:1206–11. 22382157 10.1161/STROKEAHA.111.636373

[13] Lennon LT , Ramsay SE , Papacosta O , Shaper AG , Wannamethee SG , Whincup PH . Cohort profile update: The British Regional Heart Study 1978‐2014: 35 years follow‐up of cardiovascular disease and ageing. Int J Epidemiol. 2015;44:826–6, 826g. 26232420 10.1093/ije/dyv141 PMC4521137

[14] Wannamethee SG , Lowe GDO , Whincup PH , Rumley A , Walker M , Lennon L . Physical activity and hemostatic and inflammatory variables in elderly men. Circulation. 2002;105:1785–90. 11956120 10.1161/hc1502.107117

[15] Andreescu ACM , Cushman M , Rosendaal FR . D‐dimer as a risk factor for deep vein thrombosis: the Leiden thrombophilia study. Thromb Haemost. 2002;87:47–51. 11858188

[16] Lenting PJ , van Mourik JA , Mertens K . The life cycle of coagulation factor VIII in view of its structure and function. Blood. 1998;92:3983–96. 9834200

[17] Koster T , Blann AD , Briet E , Vandenbroucke JP , Rosendaal FR . Role of clotting factor VIII in effect of von Willebrand factor on occurrence of deep‐vein thrombosis. Lancet. 1995;345:152–5. 7823669 10.1016/s0140-6736(95)90166-3

[18] Rietveld IM , Lijfering WM , Le Cessie S , Bos MHA , Rosendaal FR , Reitsma PH , et al. High levels of coagulation factors and venous thrombosis risk: strongest association for factor VIII and von Willebrand factor. J Thromb Haemost. 2019;17:99–109. 30471183 10.1111/jth.14343

[19] Wu O , Bayoumi N , Vickers MA , Clark P . ABO(H) blood groups and vascular disease: a systematic review and meta‐analysis. J Thromb Haemost. 2008;6:62–9. 17973651 10.1111/j.1538-7836.2007.02818.x

[20] Ohira T , Cushman M , Tsai M , Zhang Y , Heckbert SR , Zakai NA , et al. ABO blood group, other risk factors and incidence of venous thromboembolism: the longitudinal investigation of thromboembolism etiology (LITE). J Thromb Haemost. 2007;5:1455–61. 17425663 10.1111/j.1538-7836.2007.02579.x

[21] Lowe GD , Woodward M , Vessey M , Rumley A , Gough P , Daly E . Thrombotic variables and risk of idiopathic venous thromboembolism in women aged 45‐64 years. Relationships to hormone replacement therapy. Thromb Haemost. 2000;83:530–5. 10780311

[22] Van Hylckama VA , van der Linden IK , Bertina RM , Rosendaal FR . High levels of factor IX increase the risk of venous thrombosis. Blood. 2000;95:3678–82. 10845896

[23] Luxembourg B , Schmitt J , Humpich GM , Seifried E , Lindhoff‐Last E . Intrinsic clotting factors in dependency of age, sex, body mass index, and oral contraceptives: definition and risk of elevated clotting factor levels. Blood Coagul Fibrinolysis. 2009;20:524–34. 19620844 10.1097/MBC.0b013e32832d9b58

[24] Tripodi A , Chantarangkul V , Martinelli I , Bucciarelli P , Mannucci PM . A shortened activated partial thromboplastin time is associated with the risk of venous thromboembolism. Blood. 2004;104:3631–4. 15297315 10.1182/blood-2004-03-1042

[25] Lowe GD , Haverkate F , Thompson SG , Turner RM , Bertina R , Turpie AG , et al. Prediction of deep vein thrombosis after elective hip replacement surgery by preoperative clinical and haemostatic variables: the ECAT DVT study. Thromb Haemost. 1999;81:879–86. 10404761

[26] Lowe GDO , Rumley A , Woodward M , Reid E , Rumley J . Activated protein C resistance and the FV: R506Q mutation in a random population sample: associations with cardiovascular risk factors and coagulation variables. Thromb Haemost. 1999;81:918–24. 10404768

[27] Sveinsdottir SV , Svensson PS , Engstrom G . Inflammatory plasma markers and risk for venous thromboembolism. J Thromb Thrombolysis. 2014;38:190–5. 24307292 10.1007/s11239-013-1033-6

[28] Rosendaal FR , van Hylckama VA , Doggen CJM . Venous thrombosis in the elderly. J Thromb Haemost. 2007;5(Suppl.1):310–7. 17635742 10.1111/j.1538-7836.2007.02489.x

[29] Rumley A , Emberson JR , Wannamethee SG , Lennon L , Whincup PH , Lowe GDO . Effects of older age on fibrin D‐dimer, C‐reactive protein and other haemostatic and inflammatory variables in men aged 60‐79 years. J Thromb Haemost. 2006;4:982–7. 16689748 10.1111/j.1538-7836.2006.01889.x

